# PRMT5/Wnt4 axis promotes lymph-node metastasis and proliferation of laryngeal carcinoma

**DOI:** 10.1038/s41419-020-03064-x

**Published:** 2020-10-15

**Authors:** Nan Wang, Honghong Yan, Di Wu, Zheng Zhao, Xiaoqi Chen, Qian Long, Changlin Zhang, Xiaohao Wang, Wuguo Deng, Xuekui Liu

**Affiliations:** 1 College of Life Science, Jiaying University, Meizhou, China; 2 Sun Yat-sen University Cancer Center, State Key Laboratory of Oncology in South China, Collaborative Innovation Center of Cancer Medicine, Guangzhou, China

**Keywords:** Metastasis, Prognostic markers

## Abstract

Metastasis is the main cause of laryngeal cancer-related death; its molecular mechanism remains unknown. Here we identify protein arginine methyltransferase 5 (PRMT5) as a new metastasis-promoting factor in laryngeal carcinoma, and explore its underlying mechanism of action in regulating laryngeal cancer progression. We illustrated that PRMT5 expression was positively correlated with tumor stages, lymphatic metastasis, and unfavorable outcome. Functional assays revealed that PRMT5 promoted laryngeal carcinoma cell proliferation, migration, and invasive capacity in vitro, as well as lymph-node metastasis in vivo. The ectopic expression of PRMT5 induced EMT with downregulation of E-cadherin and upregulation of N-cadherin, snail, and MMP9. Mechanistic results revealed that the metastatic effects could be attributed to PRMT5-mediated activation of Wnt signaling, and Wnt4 is an important driver of Wnt/β-catenin signaling pathway. Wnt4 silencing could reverse PRMT5-induced cell proliferation, migration, and invasion capacities. Furthermore, inhibition of the Wnt/β-catenin signaling pathway abolished the effect of PRMT5-induced proliferation, whereas activation of the pathway enhanced the effect of PRMT5 overexpression on cell proliferation. These results demonstrated that the oncogenic role of PRMT5 could be attributed to PRMT5/Wnt4 axis-mediated activation of the Wnt/β-catenin signaling pathway. PRMT5 may serve as a novel prognostic marker and a therapeutic target for lymphatic metastasis of laryngeal carcinoma.

## Introduction

As a malignancy in the head and neck region, laryngeal carcinoma represents the second most commonly occurring cancer of the respiratory tract, >95% of which is squamous cell carcinoma. In 2018, data statistics showed that there were 177,422 new cases and 94,771 deaths worldwide^1^. Despite major advances in diagnosis and treatment, the prognosis of patients with advanced laryngeal carcinoma remains dismal, and lymph-node metastasis is the leading cause of laryngeal carcinoma-related mortality^2,3^. Nevertheless, the molecular mechanism of laryngeal carcinoma lymph-node metastasis is not yet fully understood.

Protein arginine methyltransferase 5 (PRMT5), a type II methyltransferase, can epigenetically regulate gene transcription by symmetrically dimethylated histones (e.g., H4, H3, and H2A)^4–6^ as well as nonhistone proteins (e.g., p53, E2F1, GATA4, and EGFR)^7–10^. PRMT5 is overexpressed in a variety of cancers, and its activity is associated with cell transformation. Recent evidence suggests that PRMT5 contributes to tumorigenesis. For instance, PRMT5 overexpression or hyperactivation is observed in several tumor types, including acute myeloid leukemia, breast cancer, glioblastoma, lung, and prostate cancer, which facilitates tumor initiation and progression^11–16^. PRMT5 overexpression positively correlates with astrocytoma progression, and inversely correlates with survival^13^. Kryukov et al.^17^ reported that PRMT5 repression induced tumor shrinkage and cell apoptosis. Depletion of PRMT5 in glioma cell lines failed to form tumors in mice engrafted intracranially with GBM xenografts^13^, suggesting that PRMT5 serves as a potential therapeutic target for cancer therapy. Moreover, PRMT5 affects epithelial–mesenchymal transition (EMT) and is overexpressed during oncogenesis and progression in oral squamous cell carcinoma^18^. Tarighat et al.^11^ found that PRMT5 was recruited to the snail complex via interaction with the scaffold protein AJUBA to repress E-cadherin expression, resulting in EMT process. PRMT5 also regulates cell-cycle progression by increasing the expression of positive regulators of the G1 phase (Cyclin D1, Cyclin D2, and CDK4)^19^.

EMT is a physiological process in which the epithelial cells lose their polarity and adhesion and can be converted into mesenchymal states^20^. EMT is hijacked by cancer cells to leave the primary tumor site, invading the surrounding tissue, which is frequently associated with distant metastasis and tumor dissemination^21^. EMT is induced by genetic and epigenetic changes within the transforming cells, as well as signals from the tumor microenvironment^22^. A variety of signaling pathways are involved in EMT, including nuclear factor-ĸB (NF-ĸB), transforming growth factor-β (TGF-β), Notch, and Wnt/β-catenin pathways^23^. The canonical Wnt pathway plays critical roles in developmental processes, human tumorigenesis, and metastasis^24,25^. The Wnt family consists of 19 conserved genes that encode for secreted glycoprotein ligands for Frizzled receptors^26^. Wnt4 is a member of the Wnt family, and previous reports have shown that abnormal Wnt4 expression is associated with carcinogenesis, and it regulates the proliferation of cancer stem cells in response to progesterone^27–29^.

In this study, we set out to reveal the contributions of PRMT5 in metastasis formation of laryngeal carcinoma via the Wnt/β-catenin pathway. Using both in vivo and in vitro models, we demonstrated that PRMT5 can facilitate EMT and lymph-node metastasis of laryngeal carcinoma cells via modulating the Wnt4/β-catenin pathway. PRMT5 may serve as a promising therapeutic target for highly invasive laryngeal carcinoma patients.

## Materials and methods

### Human tissue sample and cell lines

A total of 176 formalin-fixed, paraffin-embedded tissues, including primary laryngeal carcinoma, lymph-node metastatic carcinoma, and normal tissues, were collected from the Sun Yat-sen University Cancer Center of Sun Yat-sen University (Guangzhou, China) between January 2010 and December 2018. A tissue microarray consisting of 66-case laryngeal carcinoma and lymph-node metastatic carcinoma tissues was purchased from Alenabio (Xi’an, China). This study was performed in accordance with the institutional ethical guidelines and was approved by the Ethics Committee of the Sun Yat-sen University Cancer Center. Informed consent was obtained from the patients before the study began, none of whom had received chemotherapy or radiotherapy prior to surgery. The laryngeal cancer cell lines Tu212 and Tu686 were purchased from Guangzhou Juyan Biological Technology (Guangzhou, China) and cultured in RPMI 1640 (Gibco, USA). H2e, M4e, and NOK cell lines were purchased from Shanghai Institute of Life Sciences Cell Resource Center, China Academy of Sciences (Shanghai, China) and cultured in Dulbecco’s modified Eagle’s medium (Gibco, USA). All media were supplemented with 10% fetal bovine serum (FBS) (Gibco, USA) and 1% penicillin–streptomycin solution (Invitrogen, USA). Cells were grown in a humidified atmosphere of 5% CO_2_ at 37 °C. All cell lines used in this study tested negative for mycoplasma contamination.

### Immunohistochemistry staining and scoring analyses

The paraffin-embedded tissues were sectioned and then deparaffinized and rehydrated. The primary antibodies against PRMT5 (#79998) were obtained from Cell Signaling Technology (Danvers, MA, USA). Anti-Ki67 (M00254-1) was obtained from BOSTER Biotech (Wuhan, China). Wnt4 (sc-376279) was obtained from Santa Cruz Biotechnology (Santa Cruz, CA, USA). Next, the sections were probed with the secondary antibody at 37 °C for 30 min and developed by diaminobenzidine staining solution (Bioss, Beijing, China). The expression of PRMT5 was blindly quantified by two pathologists according to a staining scoring system. Five high-power visual fields were randomly selected from each slice (×200) with 100 cells per field. The PRMT5-positive expression results were determined based on their respective percentage of positive cells: number of PRMT5-positive tumor cells/number of total tumor cells ≥10% was considered to be positive (+) and <10% was considered to be negative (−).

### RNA interference and plasmid transfection

siRNA oligonucleotides targeting PRMT5, Wnt4, and negative control siRNA were purchased from Ribobio (Guangzhou, China). The siRNA sequences for PRMT5 were 5′-GCACCAGTCTGTTCTGCTA-3′ and 5′-GAGGTGCAGTTCATCATCA-3′. The sequences for Wnt4 siRNA were 5′-GCCAAGUCCAGACUUCUGUT-3′ and 5′-GCAGACGCTCTGGTTGTTA-3′. siRNA transfection used was Lipofectamine RNAimax (Life Technologies) according to the manufacturer’s instructions. After a 48-h period of transfection, the cells were collected for subsequent experimentation. The plasmids were transfected in strict accordance with the instructions of lipofectamine 3000 (Invitrogen, USA).

### RNA sequencing analysis and the database used in this study

Cells were transfected with siPRMT5 (siRNA-2) or control siRNA for 48 h. TRIzol (Invitrogen, USA) was used to extract the total RNA from laryngeal carcinoma cells and tissues. Library construction and sequencing were performed by Sinotech Genomics (Shanghai, China). The libraries were sequenced on an Illumina HiSeq 2500 platform, and 100-bp paired-end reads were generated. *ASprofile* was used to analyze the differentially alternative splicing events. Differentially expressed genes were screened by the threshold of 2.0-fold change and *p* value lower than 0.05. All RNA-seq raw data have been deposited in the GEO database (accession no. GSE154449).

The Cancer Genome Atlas (TCGA) data regarding laryngeal carcinoma patients, including genomic alterations, gene expression, and clinical information, were obtained from the TCGA Data Portal website (https://portal.gdc.cancer.gov/projects/). Microarray datasets of laryngeal carcinoma patients for gene expression analysis were acquired from online data repository (Gene Express Omnibus, GEO) dataset (GSE51985).

### Lentivirus transduction

Full-length PRMT5 and shRNA-PRMT5 were cloned, respectively, into pCDH-CMV-MCS-EF1-Puro and pEZ-Lv217.1-Luc-Puro vectors. PRMT5-shRNA oligonucleotide sequences are as follows: sh1forward: 5′-CCGGGCACCAGTCTGTTCTGCTACTCGAGTAGCAGAACAGACTGGTGCTTTTTG-3′; sh1reverse: 5′-AATTCAAAAAGCACCAGTCTGTTCTGCTACTCGAGTAGCAGAACAGACTGGTGC-3′; sh2forward: 5′-CCGGGAGGTGCAGTTCATCATCACTCGAGTGATGATGAACTGCACCTCTTTTTG-3′; sh2reverse: 5′-AATTCAAAAAGAGGTGCAGTTCATCATCACTCGAGTGATGATGAACTGCACCTC-3′. The recombinant lentivirus was produced by cotransfecting HEK293T cells with PRMT5 shRNA by using Lipofectamine 3000 (Invitrogen, Thermo Fisher Scientific). Stable cell lines were obtained after selection with 1 μg/ml puromycin (Invitrogen) for 14 days. The efficiency of PRMT5 knockdown and overexpression were evaluated using quantitative reverse transcription polymerase chain reaction and western blot.

### RNA isolation and quantitative reverse transcriptase PCR (RT-PCR)

Total RNA was extracted from the tissues and transfected cells in each group in strict accordance with the instructions of TRIzol reagent (Invitrogen, New York, CA, USA). RNA quality and quantity were assessed by Nano Drop 2000 analysis. cDNA was synthesized using the Transcriptor First Strand cDNA Synthesis kit (Roche, Basel, Switzerland) according to the manufacturer’s instructions. The SYBR green PCR kit (Roche) was used for PCR amplification. Relative gene expression quantification was determined using the 2^−∆∆Ct^ method. The housekeeping gene GAPDH was used as an internal control. The aforementioned experiments were repeated three times independently. All specific primers used for RT-PCR are listed in Table 1.

**Table 1:** Primer in this study.

Gene	Forward	Reverse
PRMT	CTGTCTTCCATCCGCGTTTCA	GCAGTAGGTCTGATCGTGTCTG
c-myc	GAGCCCCTGGTGCTCCAT	GCCTGCCTCTTTTCCACAGA
Cyclin D1	CTGGAGGTCTGCGAGGAACA	AGCTGCAGGCGGCTCTTT
E-cadherin	CTTTGACGCCGAGAGCTACAC	TTGTCGACCGGTGCAATCT
GAPDH	GCACCACCAACTGCTTAGCA	TCTTCTGGGTGGCAGTGATG
Wnt7a	CTGTGGCTGCGACAAAGAGAA	GCCGTGGCACTTACATTCC
Wnt4	AGGAGGAGACGTGCGAGAAA	CGAGTCCATGACTTCCAGGT
Wnt9	AGCAGCAAGTTCGTCAAGGAA	CCTTCACACCCACGAGGTTG

### Western blotting

Following 48 h of transfection, total protein lysates were prepared using RIPA buffer (Solarbio, Beijing, China) followed by the addition of protease inhibitor cocktail (Roche) and PMSF (Solarbio), according to the manufacturers’ instructions. The protein concentration was determined using a bicinchoninic acid Protein Assay kit (Invitrogen, Thermo Fisher). Overall, 40 μg of protein was separated by 10% SDS-PAGE and then transferred to PVDF membranes (Roche, CA, USA). After being blocked with 5% bovine serum albumin (BSA) (Solarbio, Beijing, China) for 1 h at room temperature, the membranes were incubated with primary antibodies overnight at 4 °C. Then the membranes were incubated with HRP-conjugated IgG for 1 h; immunoreactivity was detected with enhanced chemiluminescence (Thermo Fisher Scientific, Rochester, NY, USA) by ChemiDoc™ XRS+ (Bio-Rad Laboratories, Inc.). Primary antibodies used in the experiment were as follows: PRMT5 (#79998, Cell Signaling Technology), E-cadherin (#3195, Cell Signaling Technology), N-cadherin (22018-1-AP, Proteintech), β-catenin (#8480, Cell Signaling Technology), snail (#3879, Cell Signaling Technology), Lamin B1 (PB9611, BOSTER Biotech), MMP9 (#13667, Cell Signaling Technology), Wnt4 (sc-376279, Santa Cruz), c-myc (ab-32072, Abcam), Cyclin D1 (60186-lg, Proteintech), Wnt7A (sc-365665, Santa Cruz), Wnt9A (ab-125957, Abcam), and GAPDH (#2118, Cell Signaling Technology).

### Cell proliferation and colony-formation assays

Cell proliferation was detected by MTS assay. Laryngeal cancer cells were seeded in 96-well plates at 2 × 10^3^ per well for 24 h. Cell proliferation was evaluated using the CellTiter96^®^AQueousOne Solution Cell Proliferation Assay kit (Promega) according to the manufacturer’s instructions. MTS reagent (20 µl) was added to 100-µl culture medium after seeding for 24, 48, 72, and 96 h, and incubated in a CO_2_ incubator at 37 °C for 2.5 h each time. The absorbance at 490 nm was measured using a Spark^®^ multimode microplate reader (Tecan, Männedorf, Switzerland). Each experiment with five replicates was repeated three times. For colony-formation assays, 500 cells were seeded in six-well culture dishes and incubated until visible colonies (≥50 cells) formed in complete growth medium (10 days–2 weeks). Megascopic cell colonies were fixed with methanol, stained with 0.1% crystal violet (Sigma-Aldrich, St. Louis, MO), and counted manually.

### Transwell migration and invasion assays

For migration assays, Tu686 and Tu212 cells (5 × 10^4^/well) were suspended in 300 μl of serum-free medium and plated into the upper chambers (24-well insert, 8-μm pores, Corning, USA), and 500 μl of medium containing 20% FBS was placed into the lower chambers. After 24 or 36 h, the cells under the bottom membrane were stained with crystal violet. The migrating cells in five randomly selected fields at a magnification of ×200 were imaged using digital microscopy and counted. For invasion assays, the upper chamber was precoated with 50 μl of Matrigel (BD Biosciences, CA, USA); the remaining steps were as described above for the migration assay. All assays were performed in triplicate.

### Wound-healing assay

After 12 h of transfection in each group, an artificial wound was scratched with a 200-μl pipette tip. The cells were then rinsed with PBS, and then the culture medium was replaced with 1% FBS medium. The injured areas were photographed at 0 and 48 h later using a light microscope (CKX43, Olympus) at ×100. The migration of cells was assessed by the changes in the distances. Three duplicate wells were set in each group, and the experiment was repeated in triplicate.

### TOP/FOP-flash reporter assay

In order to estimate the Wnt/β-catenin activity, β-catenin/TCF was transfected according to the instructions (Millipore Billerica, MA, USA). The 5 × 10^3^ Tu212 cells were seeded into 24-well dishes. After 24 h, the cells were transiently transfected with either 2 μg of pTOP flash or pFOP flash plasmids (TCF Reporter Plasmid/mutant, inactive TCF-binding site, Millipore) and pSV40-Renilla plasmid (0.5 μg, Promega) as an internal control by using Lipofectamine 3000 (Invitrogen). Following transfection for 48 h, both Renilla and firefly luciferase were analyzed using a Dual-Luciferase Reporter Assay kit (Promega) according to the instructions, with a Glomax luminometer (Promega). The luciferase activity of each well was normalized to Renilla luciferase activity. The experiment was repeated in triplicate.

### Immunofluorescence assay

Frozen sections of tissue samples or cell slides were fixed with 4% paraformaldehyde for 20 min, permeabilized with 0.1% Triton X-100 for 10 min, blocked with goat serum for 1 h, and incubated with anti-E-cadherin (#3195, Cell Signaling Technology) and anti-β-catenin (#8480, Cell Signaling Technology) at 4 °C overnight. After washing with PBS, the sections were then incubated with Alexa Fluor^®^ 488 Conjugate (ab150117, Abcam). The nuclei of the cells in the confocal dishes were counterstained with 4′,6-diamidino-2-phenylindole (DAPI), and photographs were taken by a Zeiss LSM880 microscope (Zeiss, Germany).

### In vivo popliteal lymph-node metastasis and tumorigenesis assay

Animal experiments were approved by the Institute of Animal Care and Use Committee of Cancer center of Sun Yat-sen University. We confirm that all procedures involving animals were performed in accordance with relevant guidelines and regulations. Four- to five-week-old BALB/c nude mice were randomly assigned into two groups with five mice placed in each group. Lentivirus-transduced Tu212 cells (2 × 10^6^ cells) stably expressing firefly luciferase were inoculated into the lower-right footpads of mice. Lymphatic metastasis was monitored and imaged by the Bruker In-Vivo FX PRO System (Bruker company, Germany) every 10 days after tumor cell injection. Six weeks later, the mice were euthanized, and the number of metastatic nodules was recorded under the guidance of a microscope followed by tumor volume calculation. For tumorigenesis assay, 1 × 10^7^ cells were injected subcutaneously into the left flank of mice (*n* = 18). The tumor size was measured by using a caliper every 3 days, and the volume was calculated. After 33 days, the mice were euthanized, and tumors were surgically dissected. The tumor specimens were fixed in 4% paraformaldehyde.

### Statistical analysis

The SPSS 19.0 software (IBM Corp., Armonk, NY, USA) was used for statistical analysis. Samples were analyzed with unpaired Student’s *t* test assuming equal variances (two-tailed) between the two independent groups. All statistical analyses were performed using GraphPad Prism 6 (GraphPad Prism, San Diego, CA, USA). All data are presented as the mean ± standard deviation from three independent experiments. *p < 0.05, **p < 0.01, and ***p < 0.001 were considered statistically significant.

## Results

### High PRMT5 expression in laryngeal carcinoma is correlated with lymph-node metastasis and unfavorable prognosis

**Fig. 1:**
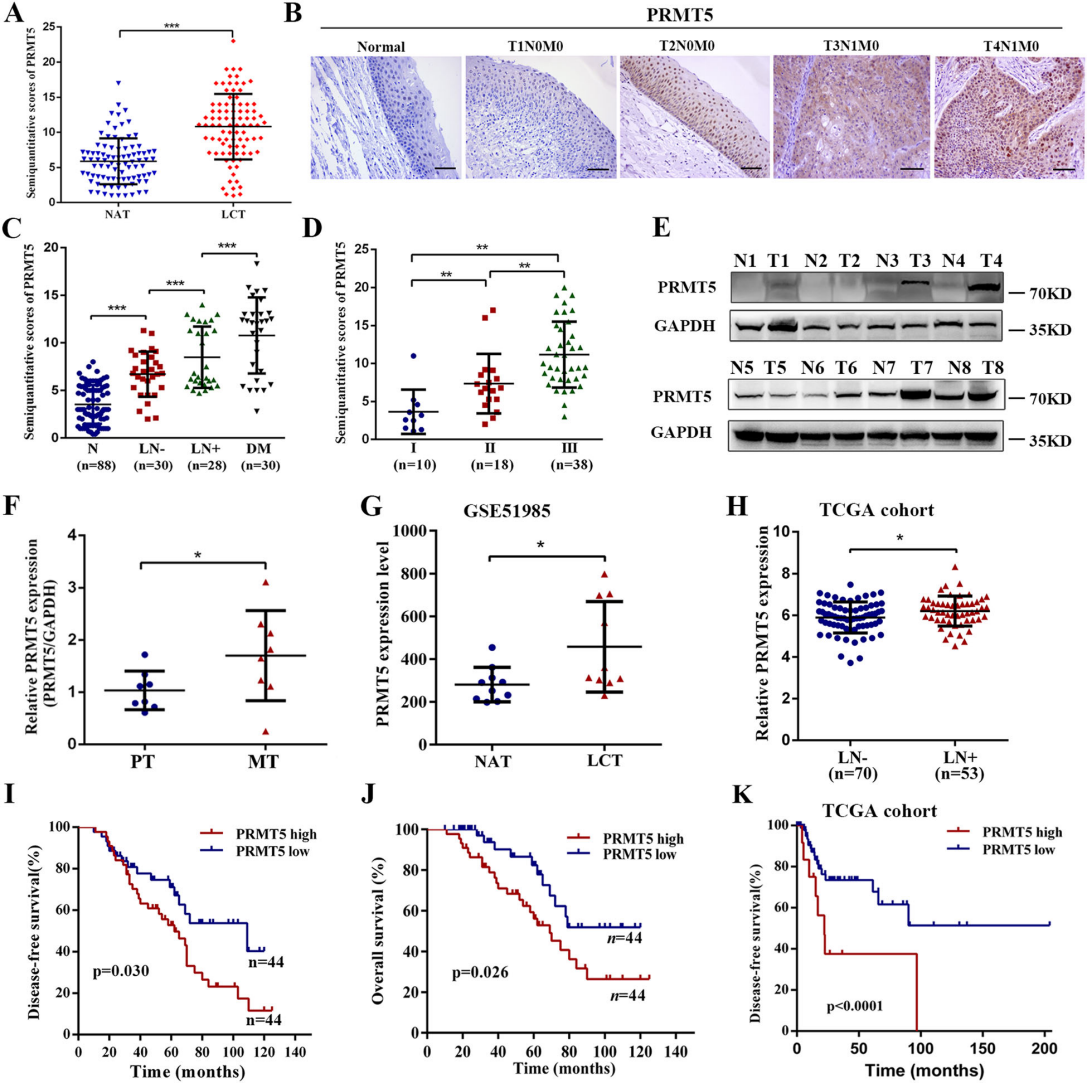
High PRMT5 expression in laryngeal carcinoma is correlated with lymph-node metastasis and unfavorable prognosis. **a** The levels of PRMT5 in 88 paired laryngeal carcinoma tissues (LCT) and normal adjacent tissues (NAT). **b** Representative IHC staining of PRMT5 expression in paraffin-embedded normal tissues and laryngeal carcinoma with or without lymph-node metastasis. Scale bar, 50 µm. **c** IHC staining statistical analysis of PRMT5 in normal tissues (N, *n* = 88), primary LCT without (LN−, *n* = 30) or with (LN+, *n* = 28) regional lymph-node and distant metastasis (DM, *n* = 30). Mean + SD. **d** IHC staining statistical analysis of the association between PRMT5 expression status and TNM stage. **e**, **f** PRMT5 expression in eight paired laryngeal carcinoma lymph-node metastasis (MT) and adjacent primary tissues (PT) was performed by western blotting and qPCR. **g** PRMT5 expression was detected in LCT and NAT from GSE51986 cohort. **h** PRMT5 expression was detected in LCT without (LN−, *n* = 70) or with (LN+, *n* = 53) lymph-node metastasis in TCGA cohort. **i**, **j** Kaplan–Meier analysis for disease-free and overall survival of laryngeal carcinoma patients with high versus low expression of PRMT5 in our cohort. **k** Kaplan–Meier curves for DFS of laryngeal carcinoma patients with high versus low expression of PRMT5 in TCGA cohort. Statistical significance was assessed using a two-tailed *t* test. *p < 0.05, **p < 0.01, ***p < 0.001.

To identify whether PRMT5 was expressed in laryngeal carcinoma and correlated with patient prognosis, a cohort of clinical samples (*n* = 88, Table S1) from laryngeal carcinoma and matched normal adjacent tissues were analyzed by immunohistochemistry. The results showed that the expression of PRMT5 was significantly higher in the laryngeal carcinoma tissues than that in the adjacent normal tissues (Fig. 1a and Supplementary Fig. S1A). PRMT5 could be exhibited in the cytoplasm, the nuclei, or both of them, as well as in high-grade laryngeal carcinoma compared with lower-grade tissues with the most prominent expression in a nuclear distribution (Fig. 1b). Notably, the PRMT5 protein level was lower in normal tissues, but higher as disease severity progressed in laryngeal carcinoma tissues without lymph-node metastasis, regional lymph-node metastasis, and distant metastasis tumor tissues (Fig. 1c). We further analyzed the correlation between PRMT5 and tumor progression by IHC on the second cohort of a 66-case tissue microarray (Table S2). The 66 patients were divided into stage I: 10 cases, stage II: 18 cases, and stage III: 38 cases. The expression of PRMT5 was elevated in stages II and III laryngeal carcinoma tissues compared to stage I tissues (Fig. 1d and Supplementary Fig. S1B). We further examined PRMT5 expression in an additional eight fresh–frozen lymphatic metastatic tumors by qPCR and western blotting. Increased expression of PRMT5 was found in lymphatic metastatic tissues compared to the corresponding primary tumor tissues (Fig. 1e, f). Subsequently, analysis of a cohort (GSE51985) from the GEO dataset revealed that PRMT5 expression was drastically higher than that in the adjacent normal patients (Fig. 1g). Moreover, a 123-case cohort from The Cancer Genome Atlas (TCGA) database showed that PRMT5 expression was upregulated in the laryngeal carcinoma with lymph-node metastasis compared with the tissues without lymph-node metastasis (Fig. 1g, h).

The heterogeneity in the degree of PRMT5 expression in different-stage tumors prompted us to examine whether PRMT5 expression index correlated with laryngeal carcinoma patients’ survival. Kaplan–Meier survival analysis showed that patients with high levels of PRMT5 expression in tumors had shorter disease-free survival (DFS) and overall survival time in our cohort (Fig. 1i, j). The association of high PRMT5 expression with poor clinical DFS outcome was confirmed by analysis of the TCGA cohort (Fig. 1k). Further, univariate and multivariate Cox regression analysis revealed that PRMT5, TNM stage, and lymph-node status may serve as an independent prognostic factor for overall survival of laryngeal carcinoma patients (Table S3). Taken together, these observations strongly suggest that PRMT5 might play a pivotal role in laryngeal carcinoma progression.

### PRMT5 is essential for the proliferation of laryngeal carcinoma cells in vitro

**Fig. 2:**
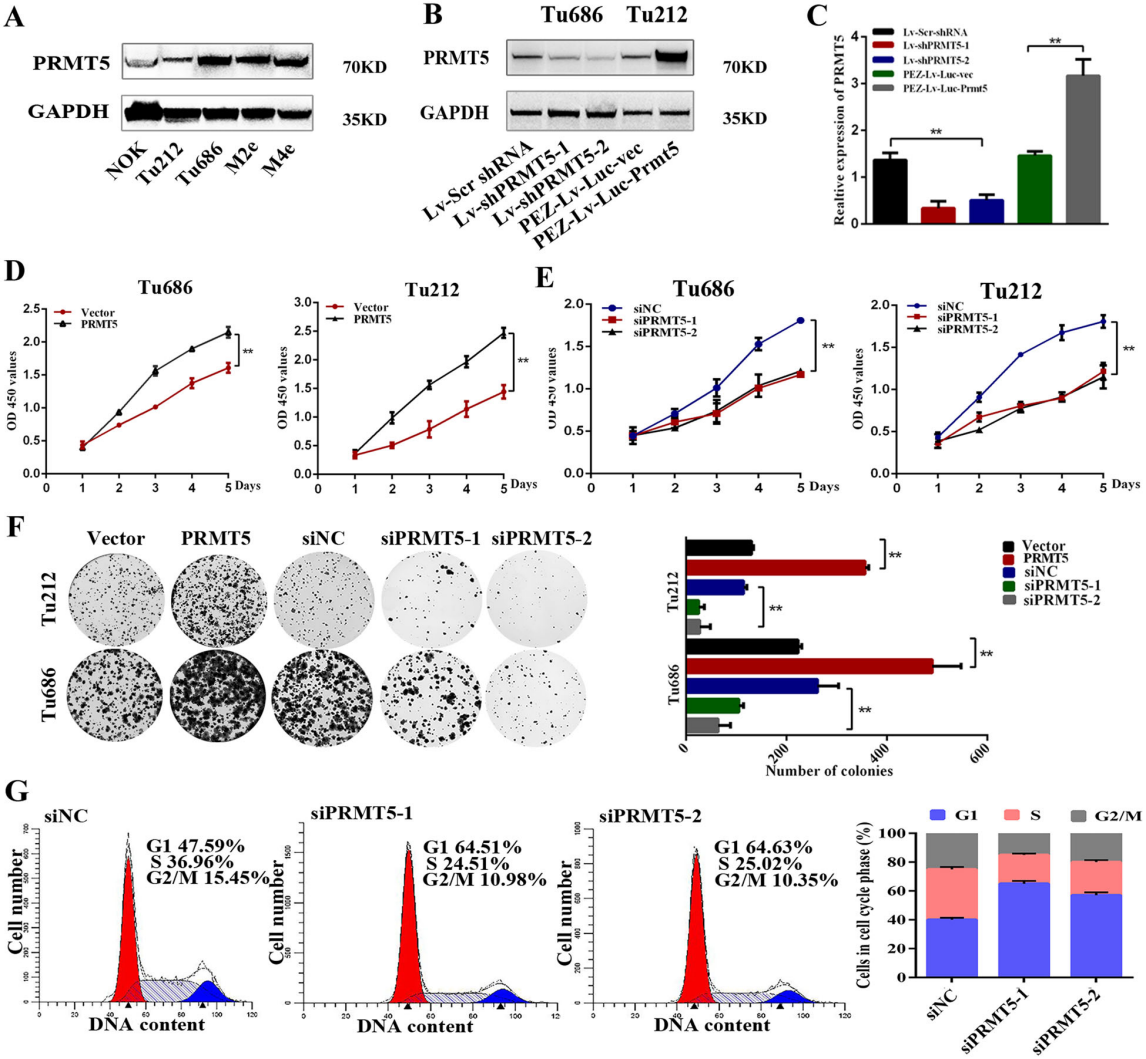
PRMT5 is essential for the proliferation of laryngeal carcinoma cells in vitro. **a** The protein levels of PRMT5 in four laryngeal carcinoma cell lines and normal oral mucosa epithelial cell were detected by western blotting. **b**, **c** Western blotting and qPCR analysis were used to confirm the efficiencies of PRMT5 in overexpression and knockdown of stable cell lines Tu686 and Tu212. **d**, **e** Effect of PRMT5 on cell viability was evaluated by CCK8 assay in Tu686 and Tu212 cells. **f** Effect of PRMT5 on colony formation in Tu686 and Tu212 cells. **g** Knockdown of PRMT5 increased the G1 fraction, as detected by flow cytometry. All the experiments were repeated three times, and the results are presented as mean ± SD. **p < 0.01.

To investigate the potential effect of PRMT5 in laryngeal carcinoma, first, we examined the expression status of PRMT5 in laryngeal carcinoma cell lines, including Tu212, Tu686, M2e, M4e, and normal oral mucosa epithelial cell (NOK). The results with anti-PRMT5 antibody demonstrated that the PRMT5 level was higher in Tu686 cells, while Tu212 cells with a lower basal level of PRMT5 (Fig. 2a). We established stable cell lines in which PRMT5 was either induced or inhibited by lentivirus-mediated transfection; western blotting and qPCR analysis were used to confirm the efficiencies of PRMT5 overexpression and knockdown (Fig. 2b, c). We then studied the effect of PRMT5 on laryngeal carcinoma cell proliferation in vitro. CCK8 and colony-formation assays revealed that overexpression of PRMT5 in Tu212 and Tu686 cells significantly increased viability and colony formation, while PRMT5 knockdown had the opposite effect (Fig. 2d–f). Next, we used flow cytometry assay to determine whether PRMT5 was involved in cell-cycle progression. Downregulated expression of PRMT-induced G1–S arrest indicated that suppression of PRMT5 inhibited laryngeal carcinoma proliferation by arresting the cell cycle in the G1 phase (Fig. 2g).

### PRMT5 promotes cancer metastasis in vitro and in vivo

**Fig. 3:**
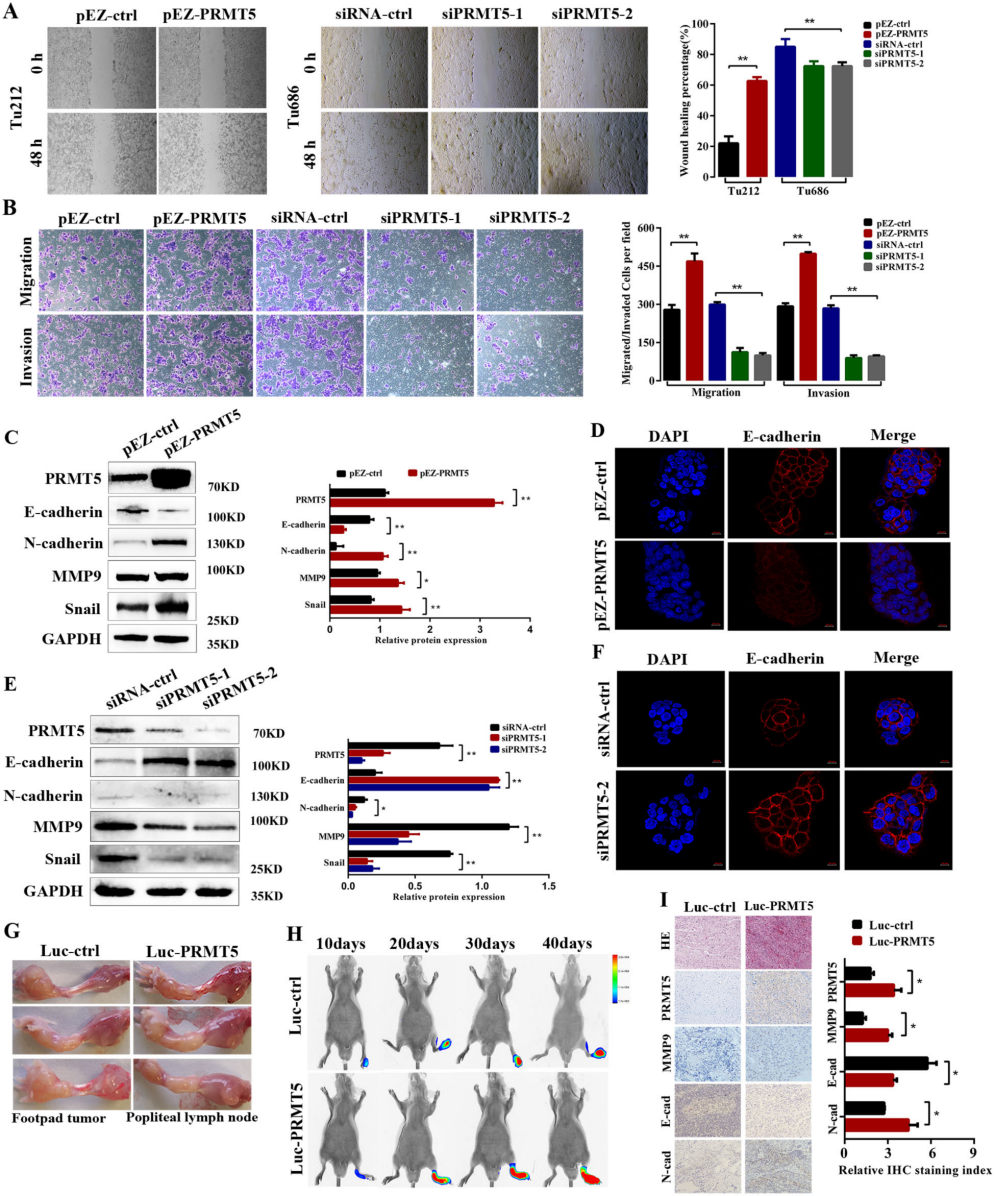
PRMT5 promotes cancer metastasis in vitro and in vivo. **a** Representative images and quantification of wound-healing assays using Tu212 and Tu686 cells showing cell mobility after overexpression or knockdown of PRMT5 (left panels); a histogram analysis of cell migration distances is shown (right panels). Scale bar, 100 µm. **b** Representative images and quantification of migration and invasion abilities in PRMT5-transduced Tu212, and PRMT5-silenced Tu686 and control cells. Scale bar, 100 µm. **c** The effect of PRMT5 overexpression on EMT marker expression was assessed by western blotting. **d** Immunofluorescence images for E-cadherin expression in Tu212 cells. **e** The effect of PRMT5 knockdown on EMT marker expression in Tu686 cells. **f** Immunofluorescence images for E-cadherin expression in Tu686 cells. Scale bar, 10 µm. **g** The nude mouse model of popliteal lymph-node metastasis. Tu212 cells stably transfected with control and PRMT5 were injected into the footpads of the nude mice, and the popliteal lymph nodes were enucleated and analyzed. **h** Bioluminescent images of primary and metastatic tumors were monitored at 10, 20, 30, and 40 days post treatment. **i** Representative images of tumor nodes were stained with H&E. The expression of PRMT5, MMP9, E-cadherin, and N-cadherin in the lymph-node metastasis model was detected by immunohistochemistry. Scale bar, 50 µm. All the experiments were repeated three times, and the results are presented as mean ± SD. Statistical significance was assessed using two-tailed *t* tests. *p < 0.05, **p < 0.01.

Since tumor cell metastasis plays a pivotal role in tumor progression, we questioned whether PRMT5 was involved in the metastasis of laryngeal carcinoma cells. To elucidate the effect of PRMT5 on the migration and mobility of laryngeal carcinoma cells, we performed in vitro wound-healing and Transwell assay, and found that PRMT5 overexpression increased migration and invasion of Tu212 cells, while PRMT5 knockdown in Tu686 cells had the opposite effect (Fig. 3a, b). These results imply that PRMT5 facilitates laryngeal carcinoma cell metastatic behavior. EMT has been reported to allow epithelial cells to acquire migratory and invasive behaviors during cancer metastasis^30^. Next, to further elucidate the biological effects of PRMT5 on laryngeal carcinoma progression, western blotting and immunofluorescent staining were used to examine the effect of PRMT5 on the expression of EMT-related markers. E-cadherin was downregulated, and N-cadherin, snail, and MMP9 were upregulated in PRMT5-overexpressed Tu212 cells compared with control (Fig. 3c). Immunofluorescence (IF) staining results showed that Tu212 cells with PRMT5 overexpressed displayed reduced epithelial marker (E-cadherin) (Fig. 3d). In contrast, the epithelial markers were upregulated, and mesenchymal markers were downregulated in Tu686 cells with PRMT5 silenced (Fig. 3e, f). Together, these results further validated that PRMT5 may play an important role in the metastatic behavior of laryngeal carcinoma in vitro.

To determine whether PRMT5 could affect laryngeal carcinoma cell invasion and metastasis in vivo, a popliteal lymph-node metastasis model was established. In total, 2 × 10^6^ laryngeal carcinoma cells (Tu212–PRMT5–Luc) that stably overexpressed PRMT5, generated by lentiviral transfection, and also stably expressed firefly luciferase, were inoculated into the footpads of nude mice (Fig. 3g). Strikingly, we discovered that Tu212–PRMT5–Luc cells effectively metastasized to the popliteal region after 6 weeks of injection, as illustrated by PET/CT bioluminescence image scanning equipment every 10 days, and calculated as the SUVmax value (Fig. 3h). Six weeks later, mice were sacrificed to examine metastatic nodules in popliteal and other organs. In addition, hematoxylin–eosin (H&E) staining and IHC results validated that PRMT5 facilitated the lymphatic metastasis of Tu212 cells. Moreover, the PRMT5-overexpression group exhibited an increase in expression of the metastasis-related marker MMP9, E-cadherin, and N-cadherin when compared with the control group (Fig. 3i). Taken together, these findings imply that PRMT5 promotes laryngeal carcinoma cell invasion and lymph-node metastasis in vivo.

### PRMT5 regulates the proliferation of laryngeal carcinoma via the Wnt signaling pathway

**Fig. 4:**
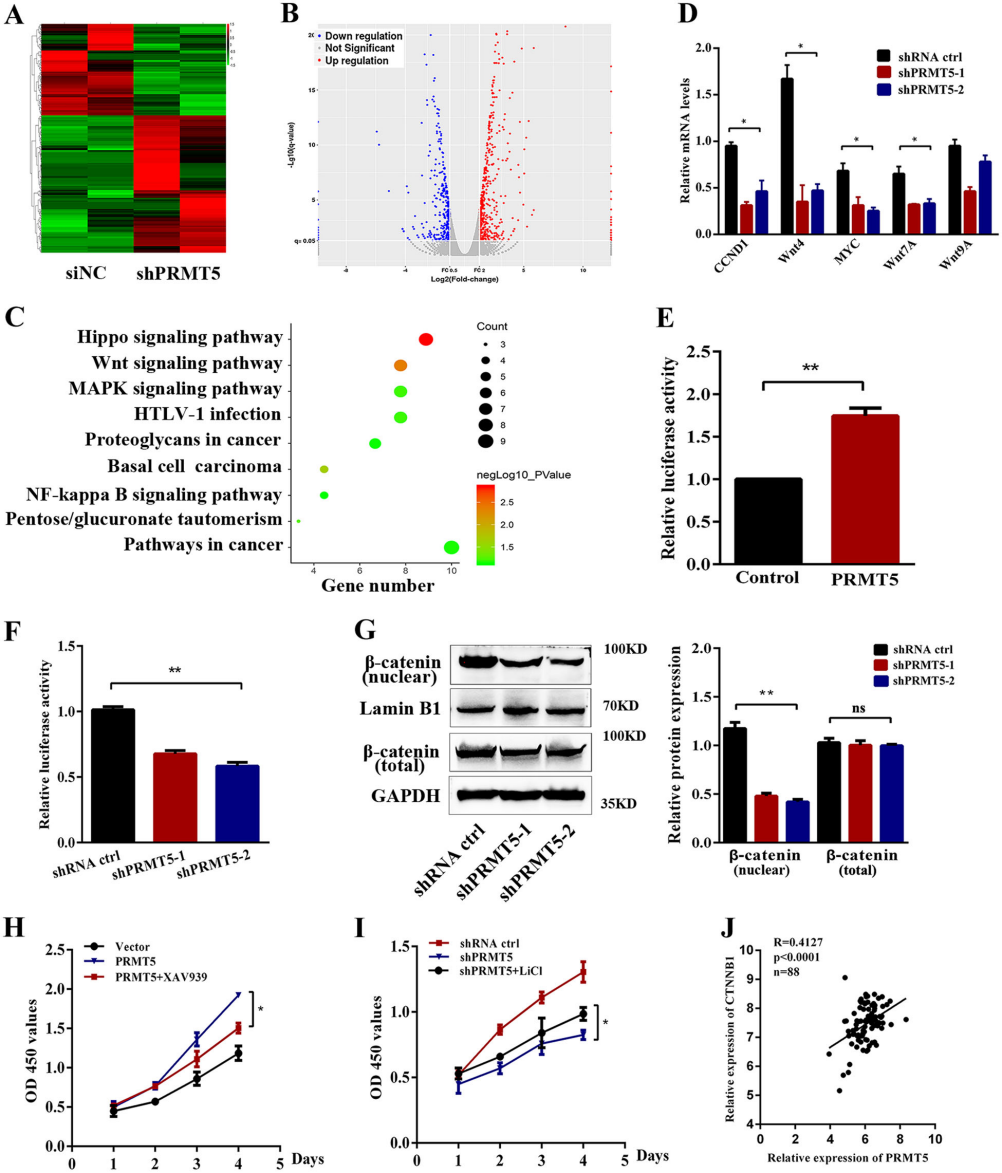
PRMT5 regulates the proliferation of laryngeal carcinoma via the Wnt signaling pathway. **a** Heatmap representing gene expression changed between shPRMT5 and shRNA ctrl cells. Red and blue indicate high and low mRNA expression, respectively. **b** Volcano plot representing gene expression differences in PRMT5-slienced Tu686 cells. Each gene is represented by a dot with the red or blue dots indicating the differentially expressed transcripts that are statistically significant. **c** KEGG enrichment analysis showed the distribution of terms exhibiting statistically significant differences. **d** The relative mRNA levels of the indicated genes were normalized to the GAPDH level in the Tu686 cells stably transfected with control or shPRMT5 as determined by qRT-PCR. The results are expressed as the mean + SD of three independent experiments. **e** Luciferase reporter assay for its activity of overexpressing PRMT5 increased Topflash reporter activities in Tu212 cells. **f** Knockdown of PRMT5 impaired Topflash reporter activities in Tu686 cells. **g** The expression of β-catenin (nuclear and total) was determined by western blotting in PRMT5-knockdown Tu686 cells. **h, i** Wnt/β-catenin pathway inhibitor XAV939 or activator LiCl dramatically suppressed or restarted cell proliferation of PRMT5 mediated, respectively. **j** Pearson correlation analysis was performed between PRMT5 and β-catenin expression in our first cohort of samples. Data are the mean ± SD of three experiments. *p < 0.05, **p < 0.01.

To gain insight into the mechanism of PRMT5-induced proliferation and metastasis of laryngeal carcinoma, we performed RNA-seq to identify transcriptional targets of PRMT5 knockdown in Tu686 and control cells. After data analysis, the mRNA expression was clustered using Cluster 3.0 software and plotted in Fig. 4a, b (fold change > 2.0, *p* < 0.05). To further explore the function of PRMT5 in gene regulation, we conducted Gene Ontology (GO) analysis to identify general functional features implemented by the PRMT5-inhibited group using downregulated genes. Kyoto Encyclopedia of Genes and Genomes (KEGG) pathway analysis demonstrated that the overlapped 721 downregulated genes were significantly associated with Hippo, Wnt, MAPK signaling pathway, and pathways in cancer (Fig. 4c). The Wnt/β-catenin pathway was broadly correlated with the process of promoting tumorigenicity and EMT induction^31,32^; we selected the Wnt signaling pathway for further studies. Next, we selected the target genes involved in the Wnt pathway, i.e., cyclin D1, Wnt4, c-myc, Wnt7A, and Wnt9A, to further confirm the results. Real-time PCR verified that downregulation of PRMT5 decreased cyclin D1, Wnt4, c-myc, and Wnt7A expression (Fig. 4d). To determine whether the Wnt/β-catenin pathway was activated by PRMT5 in laryngeal carcinoma cells, we then performed a luciferase experiment with TOP/FOP-Luc flash reporter assay. The transcriptional activity of TOP/FOP was significantly increased and reduced in Tu212 and Tu686 cells with stable overexpression or downregulation of PRMT5 (Fig. 4e, f).

Since β-catenin accumulation in the nucleus is the main event of Wnt/β-catenin pathway activation, we assessed the protein expression of β-catenin by western blotting, and found that knockdown of PRMT5 inhibited the expression of β-catenin accumulation in the nucleus, whereas the total β-catenin did not show any changes, indicating that PRMT5 might contribute to the activation of the Wnt/β-catenin pathway (Fig. 4g). Next, we used the Wnt/β-catenin pathway-specific inhibitor XAV939 or activator LiCl to treat laryngeal carcinoma cells for 96 h; the PRMT5-mediated proliferation in Tu212 and Tu686 cells was dramatically suppressed or restarted, respectively (Fig. 4h, i). Furthermore, in laryngeal carcinoma samples, PRMT5 expression was also shown to be positively correlated with β-catenin (Fig. 4j). Moreover, IHC analysis between PRMT5 and β-catenin from mice bearing PRMT5-overexpression tumors revealed that PRMT5 expression is positively correlated with β-catenin (Supplementary Fig. S2). These results suggest that PRMT5 regulated the β-catenin-dependent Wnt signaling pathway in laryngeal carcinoma cells.

### PRMT5/Wnt4 axis regulates the Wnt/β-catenin signaling pathway in laryngeal carcinoma cells

**Fig. 5:**
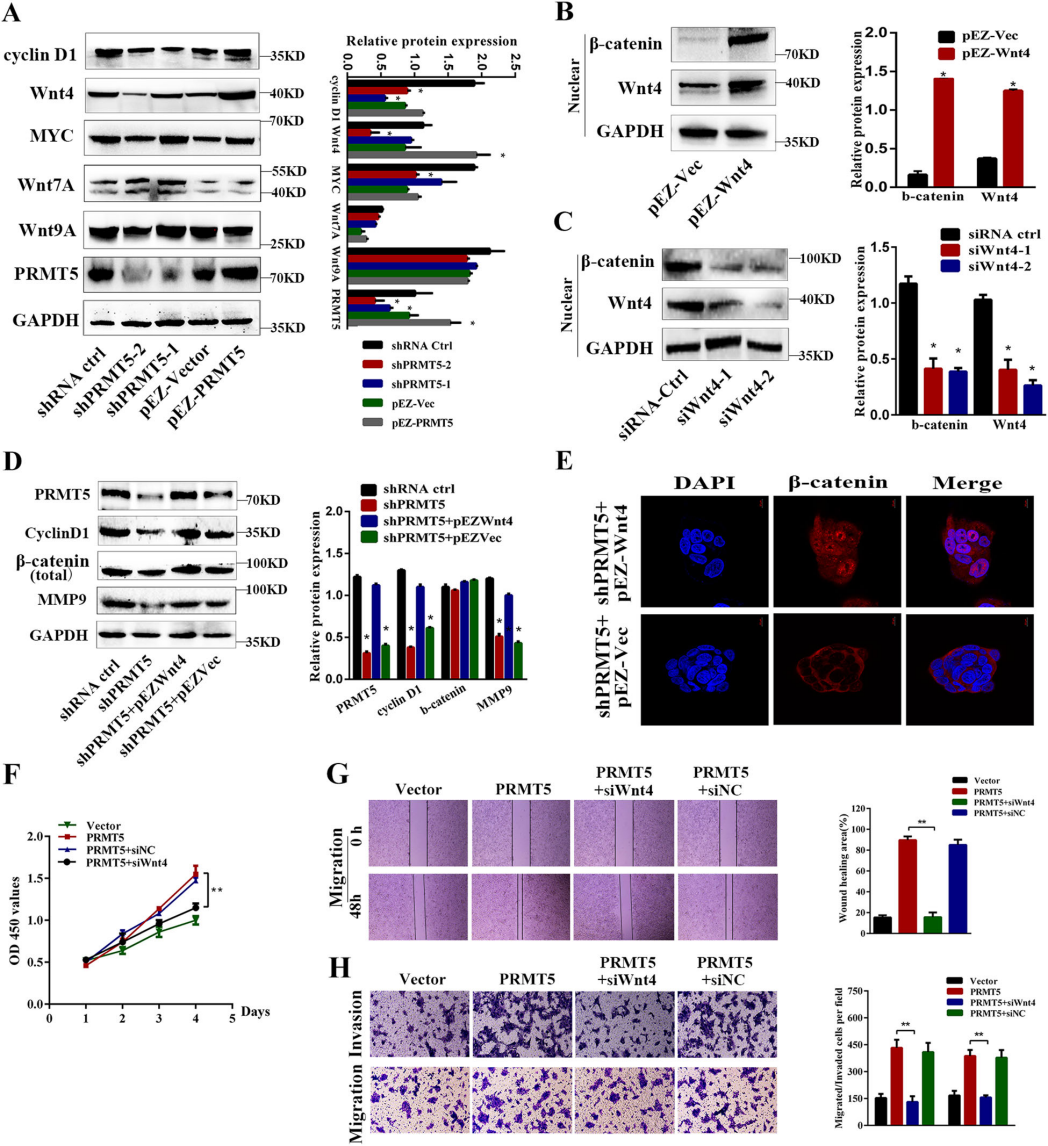
PRMT5/Wnt4 axis regulates Wnt/β-catenin signaling pathway in laryngeal carcinoma cells. **a** The expression of Wnt/β-catenin signaling pathway key proteins in PRMT5-modified Tu686 and Tu212 cells by western blotting. **b** Ectopic expression of Wnt4 in Tu212 cells increased the protein levels of β-catenin in the nucleus. **c** Depletion of Wnt4 in Tu686 cells decreased the expression of β-catenin in the nucleus. **d** Western blotting showing that the ectopic expression of Wnt4 in PRMT5-silencing cells greatly restored cyclin D1, β-catenin, and MMP9 expression. **e** Immunofluorescence assay of the increased β-catenin nuclear translocation in PRMT5-silencing or control cells combined with Wnt4 overexpression. Scale bar, 10 µm. **f**–**h** Depletion of Wnt4 reversed PRMT5-induced cell proliferation, migration, and invasion capacities. *p < 0.05, **p < 0.01.

We determine which Wnt protein was involved in the β-catenin-dependent Wnt signaling pathway induced by laryngeal carcinoma metastasis. We first examined the protein levels of the key molecular markers, including cyclin D1, Wnt4, MYC, Wnt7A, and Wnt9A, of the Wnt/β-catenin signaling pathway in laryngeal carcinoma cells. As shown in Fig. 5a, the protein levels of Wnt4, MYC, and cyclin D1 changed significantly, whereas Wnt7A and Wnt9A did not change much. Wnt4 had been reported to activate canonical Wnt signaling pathways in human pituitary adenomas, and is associated with tumor invasion in cutaneous cells^27^. We chose Wnt4 for further validation. More interestingly, there was overexpression or knockdown of Wnt4 expression in Tu212 and Tu686 cells, as shown in Fig. 5b; an intense accumulation of β-catenin in the nucleus was observed in cells transduced with Wnt4 plasmid, while knockdown of Wnt4 had the opposite results (Fig. 5c). Furthermore, overexpressed Wnt4 in PRMT5-knockdown cells abolished downregulation of cyclin D1, β-catenin, and MMP9 expression (Fig. 5d). Immunofluorescent staining results showed that the subcellular localization of β-catenin was mainly in the nucleus after overexpression of Wnt4 in PRMT5-depleted cells, which indicated that the β-catenin accumulation in the nucleus is necessary for PRMT5–Wnt4-induced laryngeal carcinoma metastasis (Fig. 5e). We further investigated whether PRMT5-induced proliferation of laryngeal carcinoma cells and metastasis was Wnt4-dependent. As shown in Fig. 5f–h, Wnt4 silencing could reverse PRMT5-induced cell proliferation, migration, and invasion capacities. These results demonstrate that Wnt4 was essential for PRMT5-mediated cell proliferation and metastasis of laryngeal carcinoma cells.

### PRMT5 facilitates tumorigenesis via Wnt4/β-catenin signaling

**Fig. 6:**
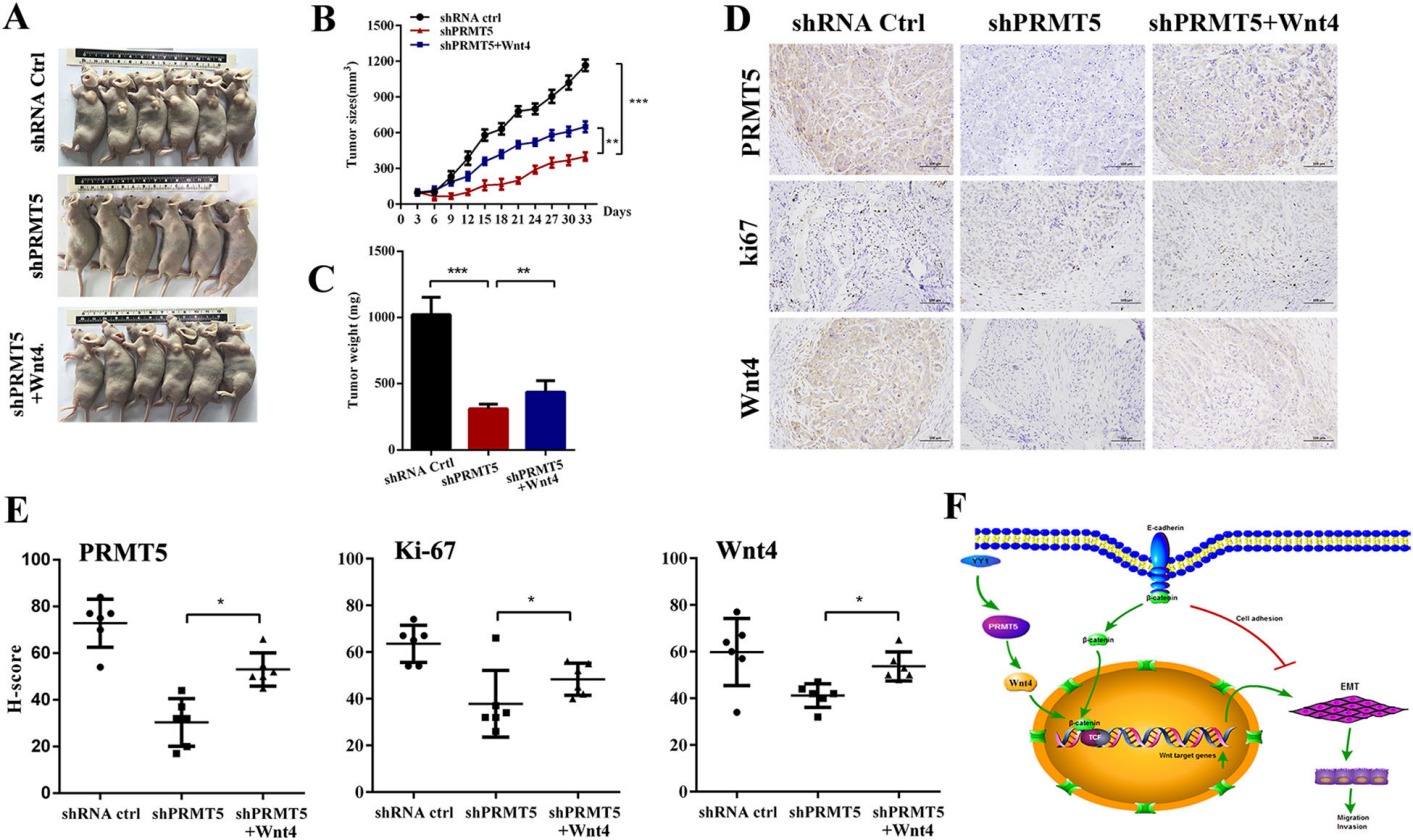
PRMT5 facilitates tumorigenesis via Wnt4/β-catenin signaling. **a** Images of the subcutaneous nude mouse model were taken in three groups. PRMT5 knockdown with Wnt4 overexpression and control group. **b** Tumor growth curves are summarized in the line chart. The average tumor volume is expressed as the mean ± SD. **c** Tumor weights were measured after the tumors were surgically dissected. **d** The PRMT5, Wnt4, and Ki67 protein expression level was detected in tumor tissues from three groups by IHC. Scale bar, 100 µm. **e** Histogram shows the *H* score of PRMT5, Wnt4, and Ki67 expression in different groups. *p < 0.05, **p < 0.01, ***p < 0.001. **f** Schematic representation of a model for the role of PRMT5 in the Wnt4/β-catenin-induced tumor metastasis in laryngeal carcinoma.

To gain insight into the impact of the PRMT5-regulated Wnt4/β-catenin pathway in vivo, we performed a nude mouse xenograft model. Tumor growth inhibition induced by knockdown of PRMT5 was restored by ectopic expression of Wnt4 (Fig. 6a). Tumor volume and weight were decreased in the PRMT5-depleted group, whereas Wnt4 overexpression could reverse PRMT5-silencing tumor shrinkage (Fig. 6b, c). Meanwhile, tumors derived from the PRMT5-knockdown group exhibited lower expression of the proliferation markers Ki67 and Wnt4 compared with shPRMT5 with the Wnt4-overexpression group (Fig. 6d, e). Collectively, these results suggested that PRMT5 regulated proliferation via the Wnt4/β-catenin axis in laryngeal carcinoma, and the PRMT5/Wnt4 axis may serve as a potential therapeutic target for laryngeal carcinoma.

In conclusion, we first reported that increased expression of PRMT5 is an unfavorable prognostic marker in laryngeal carcinoma. Moreover, by in vitro and in vivo studies, we confirm that PRMT5 is indispensable for the development of malignant laryngeal carcinoma. Mechanistic explorations showed that PRMT5 could regulate tumorigenesis and metastasis of laryngeal carcinoma by activating the Wnt4/β-catenin signaling pathway (Fig. 6f). These findings enriched our therapeutic options by blocking either PRMT5 activity or the associated transcription-regulatory network to overcome highly invasive laryngeal carcinoma and even probably other cancers.

## Discussion

Tumor recurrence and metastasis has long been the major cause of cancer death^1,33^. Over the last several decades, laryngeal carcinoma patients with early stage represent a relatively better prognosis; however, the treatment outcomes of patients with lymph-node metastasis remain frustrating. The importance of understanding the exact mechanism underlying the metastatic process has attracted considerable research interest. In this report, our findings show a correlation between PRMT5 overexpression and unfavorable prognosis of patients with laryngeal carcinoma. Mechanistically, we demonstrated for the first time that PRMT5 promotes laryngeal carcinoma metastasis through activating the Wnt/β-catenin signaling pathway.

Here we performed retrospective and prospective studies in clinical samples, and found that PRMT5 was abnormally upregulated in high-grade laryngeal carcinoma tissues. The expression of PRMT5 was positively correlated with tumor stages, lymphatic metastasis, and poor outcome. The expression of PRMT5 was significantly higher in the laryngeal carcinoma tissues than that in the adjacent normal tissues. This finding is consistent with the TCGA database and other reports in breast cancer and glioblastoma^12,13^. However, whether and how PRMT5 functions in lymph-node metastasis of laryngeal carcinoma remains unknown. Further research is needed to clarify the exact mechanisms of PRMT5 in laryngeal carcinoma invasion–metastasis. Recently, PRMT5 has been claimed as an activator of EMT in oral squamous cell carcinoma, and this could be a mechanism of PRMT5-mediated cancer invasion and metastasis^18^. Consistently, dysregulation of PRMT5 has been implicated in carcinogenesis and correlated with poor prognosis in lung and prostate cancer^15,16^. Taken together, we put forward the hypothesis that PRMT5 may serve as a biomarker for lymph- node metastasis and unfavorable prognosis in laryngeal carcinoma. In this study, we found that high PRMT5 expression was significantly correlated with worse overall survival and DFS of the patients, and showed an independent prognostic value in laryngeal carcinoma. We further demonstrated that ectopic expression of PRMT5 contributed to migration and invasion of laryngeal carcinoma cells (Tu686 and Tu212) in vitro and facilitated tumor metastasis in vivo. On the other hand, lentivirus-mediated knockdown of PRMT5 significantly inhibited cell proliferation and clonogenicity. Moreover, we observed that PRMT5 expression significantly correlated with EMT marker. Western blotting and IF analyses showed that the ectopic expression of PRMT5 induced EMT with downregulation of E-cadherin and upregulation of N-cadherin, snail, and MMP9 in laryngeal carcinoma cells. Knockdown of PRMT5 impairs its ability in downregulating E-cadherin and upregulating N-cadherin, snail, and MMP9. This is the first report on the role of PRMT5 in EMT induction.

PRMT5, a type II arginine methyltransferase, functions as a tumor initiator to regulate cancer progression by symmetric dimethylation of arginine residues on histone proteins^4,34^, and regulates important cellular functions, including cell growth, proliferation, and differentiation^35–37^. Interestingly, the subcellular localization and role of PRMT5 vary with the tumor type. In other types of tumors, the localization of PRMT5 and its target substrates in each cell chamber may be significantly different^15,38–41^. PRMT5 has been reported as an oncogene and is always complexed with MEP50/WDR77 that catalyzes arginine methylation on histones and other proteins. PRMT5 and MEP50 are required to maintain expression of metastasis and EMT markers^42^. In this study, we further elucidated the downstream signaling pathway using RNA-seq analysis; the pathway analysis of the gene expression profiling indicated that the PRMT5-mediated tumor growth and metastasis could be partially attributed to the activation of Wnt/β-catenin signaling pathway. The PRMT5-induced Wnt/β-catenin signaling was validated by TOP/FOP-flash reporter assay in Tu686 and Tu212 cells. Furthermore, the Wnt/β-catenin signaling pathway inhibitor XAV939 abolished the effect of PRMT5-induced proliferation, whereas the pathway activator LiCl enhanced the effect of PRMT5 overexpression on cell proliferation. We further found that knockdown of PRMT5 inhibited the expression of β-catenin accumulation in the nucleus, whereas the total β-catenin did not show any changes, indicating that PRMT5 might contribute to the activation of the Wnt/β-catenin pathway.

Loss of E-cadherin, a cell–cell adhesion protein responsible for intercellular attachment, is widely considered the hallmark of EMT, and is usually accompanied by deregulation of the Wnt signaling pathway. Currently, the role of EMT program in laryngeal carcinoma metastasis is poorly annotated. To gain further insight into PRMT5-mediated cancer metastasis and invasion, we investigate the downstream signaling pathway responsible for PRMT5-mediated tumor growth and metastasis, at least partly, attributed to Wnt4-increased β-catenin nuclear translocation; the induction of β-catenin signaling is critical for the proliferation and migration^29^. Meanwhile, translocation of β-catenin into the nucleus may result in the loss of E-cadherin and subsequent induction of EMT. Inhibition of Wnt/β-catenin signaling can block EMT transcription factors and promote epithelial differentiation^43,44^. Nevertheless, the mechanism underlying PRMT5- overexpression-induced EMT in laryngeal carcinoma remains unknown. In this report, we elucidate that PRMT5 overexpression can facilitate lymph-node metastasis in vivo and activate the Wnt/β-catenin signaling pathway. This was confirmed by the upregulation of β-catenin, Wnt4, cyclin D1, and MYC of the Wnt/β-catenin signaling pathway. Wnt4, a member of the Wnt family, has been shown to participate in tumor carcinogenesis^27,45^. In this study, we demonstrated that PRMT5 is a coactivator to enhance the formation of β-catenin in the nucleus of laryngeal carcinoma cells, this signaling might be at least partly mediated by Wnt4, and the PRMT5/Wnt4 axis could be a new mechanism in laryngeal carcinoma development. However, in the canonical pathway, β-catenin combined with the LEF/TCF complex to activate Wnt signaling. Further investigation is needed to indicate whether PRMT5 and LEF/TCF interacted with each other, or whether PRMT5 could form a complex with TCF/LEF in the nucleus. We thus questioned whether PRMT5 could also form a complex with β-catenin in regulating the activity of the Wnt/β-catenin pathway.

In conclusion, our present results identified the novel discovery that PRMT5 clinically and functionally participates in lymph-node metastasis and proliferation of laryngeal carcinoma by enhancing the Wnt/β-catenin pathway. Uncovering the precise role of PRMT5/Wnt4/β-catenin in the progression of laryngeal carcinoma will not only increase our knowledge of PRMT5-induced tumorigenesis, but also accelerate the development of novel biomarkers and therapeutic strategies for laryngeal carcinoma patients with lymphatic metastasis.

## Supplementary information

Supplementary tables

Supplementary figure S1

Supplementary figure S2

Supplementary figure legends
