# Supplementary material for: PRMT5/Wnt4 axis promotes lymph-node metastasis and proliferation of laryngeal carcinoma
Source: Cell Death Dis. 2020 Oct 15;11(10):864. doi: 10.1038/s41419-020-03064-x (PMC7566595; doi:10.1038/s41419-020-03064-x)
Supplement: Supplementary file 1 — Supplementary tables [file 41419_2020_3064_MOESM1_ESM.docx]

| Table S1. Correlation between clinical characteristics and PRMT5 expression of laryngeal carcinoma | | | | |
| --- | --- | --- | --- | --- |
| Characteristics | Number  (n) | PRMT5 expression  High（%） Low（%） | χ^2^ | *p* value |
| Age(years)  <50  >50 | 42  46 | 26 16  32 14 | 0.573 | 0.449 |
| Gender  Male  Female | 55  33 | 32 23  15 18 | 1.343 | 0.247 |
| Tumor stage  T1-T2  T3-T4 | 24  64 | 11 13  45 19 | 4.520 | **0.034** |
| TNM stage  I/II  III/IV | 40  48 | 16 24  30 18 | 4.427 | **0.035** |
| Differentiation  Poor/Moderate  Well | 39  49 | 17 22  30 19 | 2.714 | 0.099 |
| Lymph node metastasis  Yes  No | 58  30 | 37 21  10 20 | 7.372 | **0.007** |

**The bold ﬁgure in tables means p-value is signiﬁcant**

Table S2. Detailed information of tissue array panel

| No. | Age | Gender | Grade | TNM | Stage |
| --- | --- | --- | --- | --- | --- |
| A1 | 45 | M | 1 | T4N0M0 | III |
| A2 | 64 | M | 1 | T4N0M0 | III |
| A3 | 49 | M | 1 | T4N0M0 | III |
| A4 | 47 | M | 1 | T4N0M0 | III |
| A5 | 64 | M | 1 | T4N0M0 | III |
| A6 | 58 | M | 1 | T2N0M0 | II |
| A7 | 50 | M | 1 | T1N0M0 | I |
| A8 | 61 | M | 1 | T2N0M0 | II |
| A9 | 51 | M | 1 | T2N0M0 | II |
| A10 | 71 | M | 1 | T4N1M0 | III |
| B1 | 65 | M | 1 | T4N0M0 | III |
| B2 | 45 | M | 1 | T2N0M0 | II |
| B3 | 63 | M | 1 | T2N0M0 | II |
| B4 | 41 | M | 1 | T3N0M0 | III |
| B5 | 72 | M | 1 | T1N0M0 | I |
| B6 | 50 | M | 1 | T4N0M0 | III |
| B7 | 67 | M | 2 | T1N0M0 | I |
| B8 | 68 | M | 2 | T4aN0M0 | III |
| B9 | 72 | M | 2 | T3N0M0 | III |
| B10 | 59 | M | 2 | T4N0M0 | III |
| C1 | 48 | M | 2 | T1N0M0 | I |
| C2 | 67 | M | 1 | T4N1M0 | III |
| C3 | 54 | M | 2 | T2N1M0 | III |
| C4 | 58 | M | 2 | T2N0M0 | II |
| C5 | 71 | M | 1--2 | T3N0M0 | III |
| C6 | 64 | M | 2 | T4N1M0 | III |
| C7 | 70 | M | 3 | T3N0M0 | III |
| C8 | 75 | M | 2 | T3N0M0 | III |
| C9 | 62 | M | 2 | T3N0M0 | III |
| C10 | 63 | M | 2 | T3N0M0 | III |
| D1 | 54 | M | 2 | T4N1M0 | III |
| D2 | 64 | M | 3 | T1N0M0 | I |
| D3 | 68 | M | 2 | T3N0M0 | III |
| D4 | 53 | M | 3 | T4N1M0 | III |
| D5 | 58 | M | 3 | T2N0M0 | II |
| D6 | 72 | M | 3 | T2N1M0 | III |
| D7 | 73 | F | 3 | T3N0M0 | III |
| D8 | 47 | M | 1 | T1N0M0 | I |
| D9 | 64 | M | 3 | T3N0M0 | III |
| D10 | 47 | M | 3 | T2N0M0 | II |
| E1 | 37 | F | 2 | T4N1M0 | III |
| E2 | 40 | M | 2 | T2N0M0 | II |
| E3 | 41 | M | 2 | T4N0M0 | III |
| E4 | 54 | M | 2 | T3N0M0 | I |
| E5 | 64 | M | 2 | T4N0M0 | II |
| E6 | 49 | M | 2 | T2N1M0 | III |
| E7 | 56 | M | 2 | T4N0M0 | I |
| E8 | 69 | F | 1 | T2N0M0 | III |
| E9 | 69 | M | 3 | T4N0M0 | II |
| E10 | 52 | F | 1 | T1N0M0 | III |
| F1 | 55 | M | 2 | T3N0M0 | III |
| F2 | 52 | F | 2 | T2N0M0 | I |
| F3 | 64 | M | 2 | T3N0M0 | III |
| F4 | 68 | M | 3 | T3N0M0 | II |
| F5 | 47 | M | 3 | T1N0M0 | III |
| F6 | 70 | M | 2 | T2N0M0 | I |
| F7 | 57 | F | 3 | T3N0M0 | III |
| F8 | 64 | M | 2 | T2N0M0 | II |
| F9 | 40 | M | 3 | T4N0M0 | III |
| F10 | 77 | M | 3 | T4N1M0 | III |
| G1 | 47 | M | 2 | T3N0M0 | II |
| G2 | 44 | F | 3 | T3N0M0 | II |
| G3 | 66 | M | 3 | T4N1M0 | II |
| G4 | 62 | M | 3 | T3N0M0 | III |
| G5 | 57 | M | 2 | T3N0M0 | II |
| G6 | 74 | M | 3 | T2N0M0 | II |

Table S3. Univariate and multivariate analysis for overall survival in 88 laryngeal carcinoma cases

| Variables | Univariable  HR (95% CI) *p* value | Multivariable  HR (95% CI) *p* value |
| --- | --- | --- |
| Age(years)  <50 vs >50 | 1.361(0.878-2.156) 0.564 | 0.761(0.560-1.709) 0.358 |
| Gender  Male vs Female | 1.251(0.391-4.012) 0.693 | 1.102(0.223-3.241) 0.801 |
| TNM stage  I/II vs III/IV | 3.013(1.766-5.476) **0000** | 2.250(1.343-4.395) **0.009** |
| Tumor stage  T1-T2 vs T3-T4 | 1.813(1.103-2.967) **0.011** | 0.921(0.701-1.327) 0.402 |
| Lymph node metastasis  Yes vs No | 1.401(1.132-2.127) **0.030** | 1.327(1.036-1.843) **0.046** |
| Histology grade  Well/Moderate vs Poor | 1.680(0.968-2.781) 0.061 | 1.281(0.724-2.119) 0.272 |
| PRMT5  Low vs High | 3.011(1.817-5.048)  **0000** | 2.112(1.213-3.821) **0.011** |

**The bold ﬁgure in tables means p-value is signiﬁcant**
