# Supplementary figures and images for: PRMT5/Wnt4 axis promotes lymph-node metastasis and proliferation of laryngeal carcinoma
Source: Cell Death Dis. 2020 Oct 15;11(10):864. doi: 10.1038/s41419-020-03064-x (PMC7566595; doi:10.1038/s41419-020-03064-x)

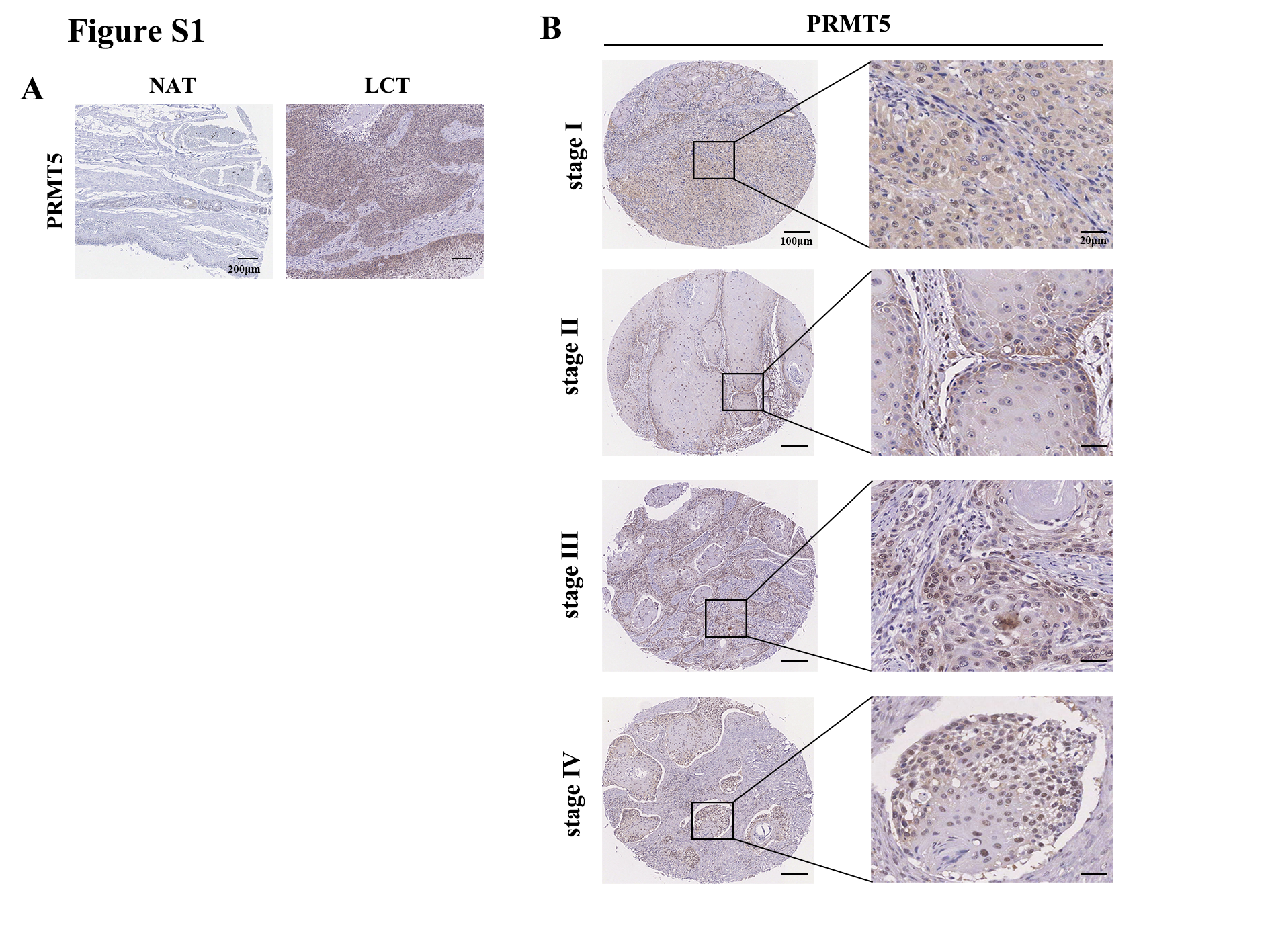

Supplement: Supplementary file 2 — Supplementary figure S1 [file 41419_2020_3064_MOESM2_ESM.tif]

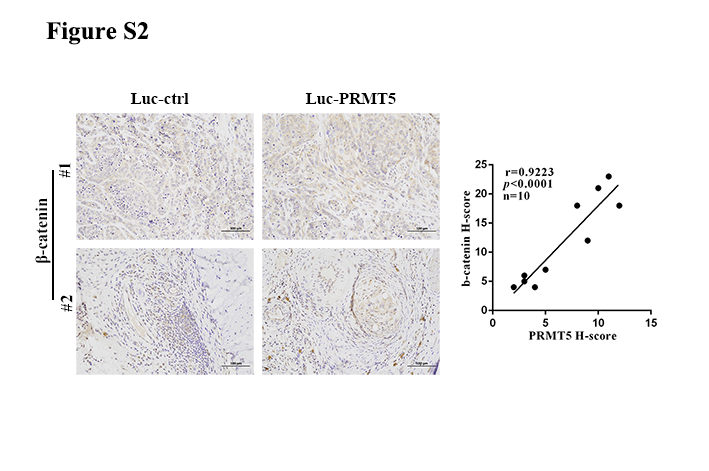

Supplement: Supplementary file 3 — Supplementary figure S2 [file 41419_2020_3064_MOESM3_ESM.tif]
