# Supplementary material for: PRMT5/Wnt4 axis promotes lymph-node metastasis and proliferation of laryngeal carcinoma
Source: Cell Death Dis. 2020 Oct 15;11(10):864. doi: 10.1038/s41419-020-03064-x (PMC7566595; doi:10.1038/s41419-020-03064-x)
Supplement: Supplementary file 4 — Supplementary figure legends [file 41419_2020_3064_MOESM4_ESM.docx]

**Supplementary figure 1. PRMT5 is highly expressed in laryngeal carcinoma.** (A) The expression of PRMT5 in laryngeal carcinoma tissues and the adjacent normal tissues. (B) The expression of PRMT5 in stage I, stage II and stage III laryngeal carcinoma tissues.

**Supplementary figure 2.** **PRMT5 exhibits positive correlation with β-catenin in xenograft model tumors.** Pearson correlation analysis was performed between PRMT5 and β-catenin expression from mice bearing PRMT5-overexpression tumors. Left panel was representative images and right panel was H-score of PRMT5 and β-catenin.
